# Crossbreeding a Mediterranean Local Chicken Breed with a Medium-Growing Commercial Hybrid as a Complementary Strategy for Conservation and Productive Valorization: Growth Performance, Carcass Traits, and Meat Quality

**DOI:** 10.3390/ani16162541

**Published:** 2026-08-14

**Authors:** Laura Martínez-Martínez, Antonio Vallecillos, Juan Orengo, Salvador Escobar, Achille Schiavone, Eva Armero

**Affiliations:** 1 Department of Agricultural Engineering, Universidad Politécnica de Cartagena Member of European University of Technology EUT+, Paseo Alfonso XIII 48, 30203 Cartagena, Spain; antonio.vallecillos@upct.es (A.V.); achille.schiavone@unito.it (A.S.); eva.armero@upct.es (E.A.); 2 Department of Animal Production, Faculty of Veterinary Science, University of Murcia, 30100 Murcia, Spain; jorengo@um.es; 3 Technical Direction, Avícola Levantina S.A., Carretera F-14, Km1, El Jimenado, 30708 Murcia, Spain; salvadorescobar@pujante.com; 4 Department of Veterinary Sciences, University of Turin, Largo Braccini 2, 10095 Grugliasco, TO, Italy

**Keywords:** crossbreeding, local breed, productive performance, valorization, conservation

## Abstract

Local chicken breeds are valuable genetic resources, but their limited growth performance may restrict practical use. This study evaluated whether crossing the Murciana chicken, a Mediterranean local breed, with the medium-growing commercial hybrid Hubbard JA757 could improve performance while retaining differentiated meat-quality traits. A total of 179 male chickens were assigned to three genotypes: Murciana (Mur), Murciana × Hubbard JA757 (MurHb), and Hubbard JA757 (Hb), reared under identical conditions, and slaughtered at a common target body weight. Final body weight was comparable among genotypes but reached at different ages (126, 78, and 53 days for Mur, MurHb, and Hb). Compared with Mur, MurHb showed higher average daily gain (30.6 vs. 21.5 g/d), improved feed conversion ratio (2.58 vs. 3.89), obtained higher carcass yield, and increased breast yield, although performance remained below Hb. Mur showed lower drip and cooking losses, whereas MurHb generally showed intermediate responses. In thigh meat, MurHb showed higher lipid content than Mur and a differentiated fatty acid profile, with lower SFA than Mur but PUFA, n-3, n-6/n-3, and PUFA/SFA values comparable to Mur and distinct from Hb. Overall, Murciana-derived crossbred improved productive performance while preserving local-breed meat-quality and lipid-related traits, supporting their potential as a complementary conservation-through-use strategy.

## Simple Summary

Local chicken breeds are part of agricultural biodiversity, but many of them grow slowly and are difficult to adapt into current poultry production systems. One possible way to support their conservation is to increase their productive value while maintaining some of their distinctive characteristics. In this study, we evaluated chickens obtained by crossing the Murciana breed, a local Mediterranean chicken breed, with a medium-growing commercial meat chicken. The aim was to determine whether this cross could improve growth and carcass performance while retaining some of the meat-quality traits observed in the local breed. The crossbred chickens grew faster and reached slaughter weight earlier than pure Murciana chickens, and they also showed better carcass yield and breast development. However, they did not become equivalent to the commercial chickens. Several meat-quality traits, including texture and the profile of fat in the thigh meat, remained closer to the Murciana breed. These results suggest that carefully designed crossbreeding may help make local chicken breeds more useful for alternative poultry production systems. This approach could support farmers, preserve animal genetic resources, and contribute to the conservation of local breeds through their practical use.

## 1. Introduction

Poultry meat is the most widely produced and consumed animal protein globally, and its demand is expected to continue increasing due to its affordability, nutritional value, and production efficiency [1,2]. This global expansion has been largely supported by the intensive use of a limited number of highly selected commercial genotypes, enabling substantial gains in productivity and feed efficiency. At the same time, this structural specialization has contributed to a progressive reduction in the use of local breeds and a narrowing of the overall genetic base of poultry production systems, raising concerns regarding long-term resilience and sustainability [3,4].

Local chicken breeds represent a valuable reservoir of animal genetic resources, playing a key role in maintaining within-species genetic diversity and preserving adaptive traits shaped under specific environmental and management conditions. These populations often exhibit rusticity, robustness, environmental resilience, and suitability for low-input or alternative production systems, together with distinctive meat quality attributes that may support differentiated market strategies [5,6]. Nevertheless, their low growth rate, reduced feed efficiency, and limited carcass yield constrain their competitiveness when compared with modern production systems, thereby compromising their economic viability and long-term conservation [7].

Conservation strategies for local chicken breeds have traditionally relied on in situ preservation and product valorization through alternative or extensive systems [8,9]. Although essential for safeguarding genetic diversity, these approaches often provide limited economic incentives for farmers and are insufficient to ensure the inclusion of local breeds under current market conditions [10]. Consequently, complementary strategies that combine conservation goals with measurable improvements in productive performance are increasingly considered within conservation-through-use frameworks [8]. These approaches combine the preservation of genetic diversity with targeted improvements in productivity and management, ensuring that local breeds remain both biologically valuable and economically sustainable under practical production conditions, in line with international frameworks that promote the sustainable use of animal genetic resources [11].

Successful examples of genotype-driven product differentiation have long been implemented in poultry production, particularly through quality-oriented schemes based on specific slow-growing lines, such as Label Rouge systems, which have consolidated stable market niches supported by defined production standards and perceived meat quality [12]. However, unlike in other livestock species, the explicit use of crossbreeding between local and commercial lines as a product-defining strategy remains comparatively less developed in poultry. In contrast, in species such as pigs, well-established market products are directly linked to specific crossbreeding schemes (e.g., Duroc × Iberian) [13]. In poultry production, crossbreeding has traditionally been applied as one of the available genetic approaches to improve productive performance, mainly through increases in growth rate, feed efficiency, or carcass yield [14]. In this context, crosses between local breeds and commercial genotypes may represent a practical strategy to obtain animals with improved productive traits that remain suitable for extensive or low-input systems, while providing meat with characteristics different from those of conventional broilers and increasingly appreciated by consumers [15]. Several studies have evaluated crossbreeding schemes involving local chicken breeds and commercial lines, reporting variable outcomes depending on the genetic background of the parental stocks and the specific production objectives to be achieved [16]. In many cases, such crosses have resulted in improvements in growth and carcass performance while retaining differentiated meat quality characteristics, [7,14,17,18]. In other cases, productive advantages were more limited [19,20], emphasizing the importance of defining clear production targets and selecting appropriate commercial genotypes when designing crossbreeding schemes linked to conservation and product differentiation [21,22]. Furthermore, cross direction represents an additional factor to consider when designing these breeding schemes. Many previous studies have evaluated local × commercial crossbreeding using a single mating direction [7,18,23,24], whereas others have specifically compared reciprocal crosses [14,19,25]. Available evidence indicates that cross direction may influence offspring performance depending on the parental combination and the traits evaluated. When natural mating is used, practical compatibility between parental genotypes should also be considered when defining the direction of the cross.

Within this framework, the Murciana chicken, a native breed from southeastern Spain belonging to the Mediterranean poultry lineage, represents a particularly relevant case study. The breed is currently maintained under an official conservation and improvement program [26] and, following several years of recovery efforts, its registered population has progressively increased, reflecting renewed interest in this conservation and productive valorization. These efforts have included specific conservation and meat valorization initiatives, such as ECOGAMUR and GENEGAMUR projects, and the recognition of purebred Murciana products under the official label “100% Murciana Autochthonous Breed”. Its rusticity, adaptation to Mediterranean conditions, and suitability for extensive and semi-extensive systems may confer advantages in alternative production models that seek to combine environmental sustainability, animal welfare, and productive resilience [27,28]. However, its limited productive performance constrains its broader economic competitiveness, despite these valuable adaptive traits. Although local × commercial hybrid crossbreeding has been evaluated in several chicken populations, no previous study has assessed this strategy in the Murciana breed. In fact, and to the best of our knowledge, this strategy has not been reported for other Spanish chicken breeds. Thus, evaluating its response to crossbreeding with a commercial genotype with slow-to-medium growth potential may offer a balanced strategy to enhance productivity while maintaining part of the structural and qualitative identity of the local breed.

Therefore, the aim of this study was to assess whether crossbreeding the Murciana chicken breed with a medium-growing commercial hybrid improves productive performance relative to the local breed while preserving key structural and qualitative traits. The main hypothesis was that this cross would improve growth performance and carcass yield compared with the local breed, while partially retaining some of its carcass traits and meat quality characteristics.

## 2. Materials and Methods

### 2.1. Ethical Statement

The experiment was conducted at the Tomás Ferro Experimental Farm of the Technical University of Cartagena (UPCT, Spain). All experimental procedures were approved by the Ethical Committee of the UPCT (expedient No. CEI23_004) and the Regional Government of Murcia (expedient No. A13260301).

### 2.2. Experimental Design and Animal Management

The experiment was conducted at the Murciana chicken nucleus facilities of the Technical University of Cartagena (UPCT, Cartagena, Spain). Three genetic groups of offspring were evaluated: the local Murciana breed (Mur), a medium-growing hybrid commercial line (Hubbard JA757; Hb), and their crossbred (Mur × Hb; MurHb). The crossbred corresponded to first-generation (F1) offspring obtained by mating Mur males with Hb females. This cross direction was selected because the lower body weight of Mur males was considered more compatible with Hb females and reduced the risk of injury under natural mating conditions.

**Figure 1:**
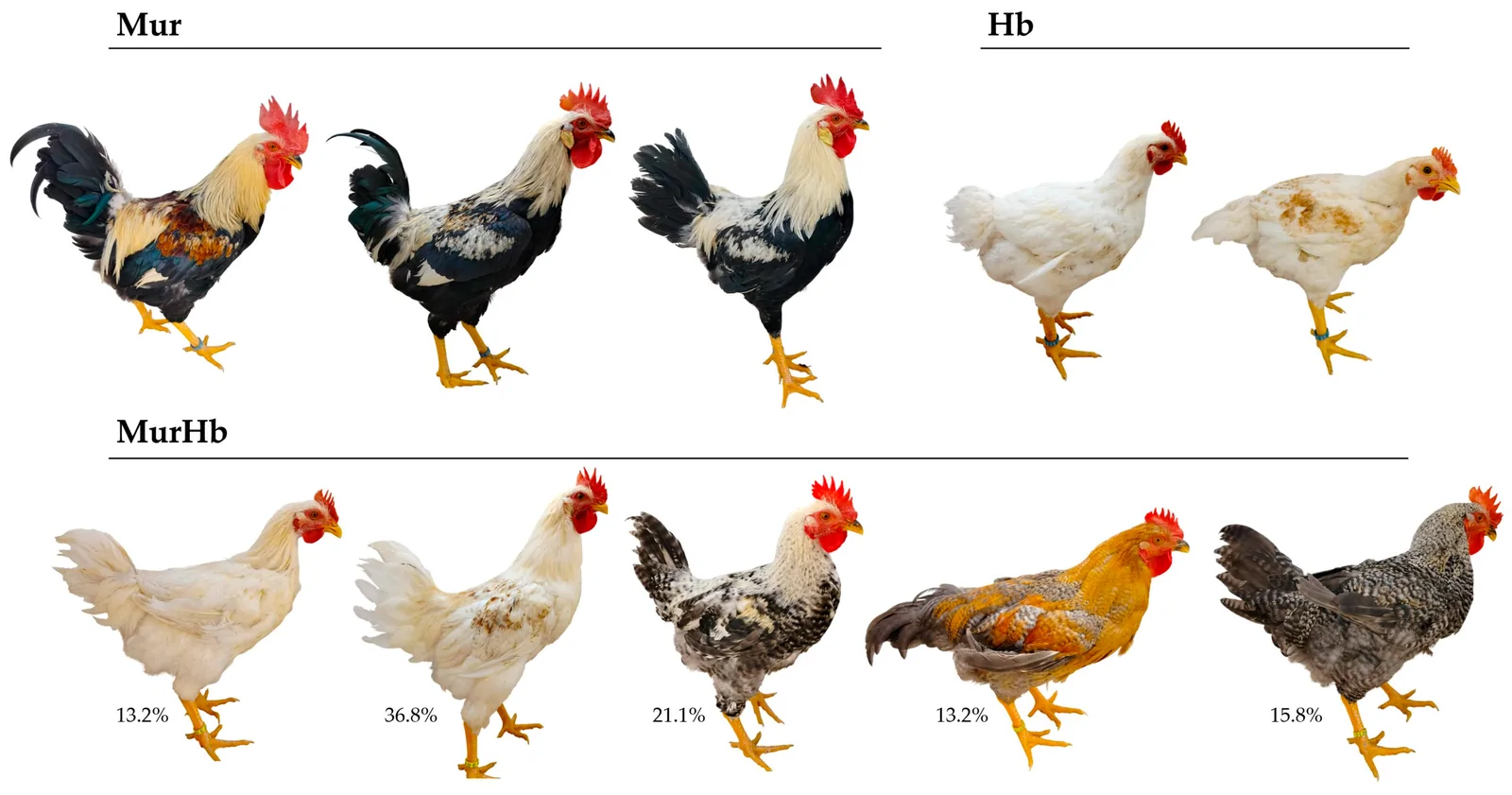
Representative pre-slaughter appearance of male chickens from the Murciana breed (Mur), the Hubbard JA757 commercial hybrid (Hb), and their F1 crossbred group (MurHb). Percentages indicate the frequency of each MurHb plumage phenotype among the 38 crossbred males photographed.

Mur and MurHb chicks were raised at UPCT facilities. For Mur reproduction, five breeding pens were established, each consisting of one male and seven females. For MurHb production, Hb females were reared at UPCT until sexual maturity, and subsequently mated with Mur males following the same breeding scheme. From breeders, eggs were collected daily, identified by breeding pen, and stored under controlled conditions (17 °C) for up to 15 days prior to incubation. Incubation was performed in an automatic incubator (Masalles 2600-I HLC, Masalles Comercial, Barcelona, Spain) at 37.7 ± 0.1 °C and 50% relative humidity during the first 19 days, with automatic turning every 45 min. From day 19 onwards, egg turning was stopped and relative humidity was increased to 60% until hatching. On the same day that Mur and MurHb chicks were hatched, Hb males were supplied at one day of age. Chicks were sexed at two weeks of age, and only males were included in the present study. Finally, a total of 54 Mur, 54 MurHb, and 66 Hb male chickens were considered. Mortality was recorded throughout the rearing period. The three genotypes were reared separately throughout the entire experimental period. Chickens were slaughtered when they reached a common target live weight of approximately 2.7 kg, selected to allow comparison among genotypes at a similar and commercially relevant body size, which resulted in different ages among genotypes. Representative images of birds from the two parental lines and the resulting crossbred genotype, taken before slaughter, are shown in Figure 1.

During the first 15 days of life, chicks were housed in an indoor brooding enclosure consisting of a heated enclosed area (1.2 m^2^) under controlled temperature conditions using a 250 W infrared lamp, connected to an adjacent exercise area (2 m^2^). Subsequently, birds were transferred to the final rearing modules, where they remained under natural environmental conditions (lighting, temperature, and ventilation) until slaughter. Each module consisted of a covered area of 5 m^2^ (5.4–6.6 birds/m^2^), equipped with feeders and drinkers, connected to an outdoor area of 24 m^2^ (1.1–1.4 birds/m^2^). Chickens had ad libitum access to water and fed. The feeding program consisted of four consecutive dietary phases: starter, early grower, late grower, and finisher. Diets were formulated and supplied by the collaborating company according to a patented feeding program designed to produce omega-3-enriched chicken meat, with enrichment mainly implemented during the finisher phase (expedient No. ES 3 020 934 B2). Diets were offered sequentially according to the commercial schedule established for the Hb line, which was used as the reference. The same dietary sequence was applied to Mur and MurHb chickens, with phase transitions adjusted to their genotype-specific growth trajectories under identical management conditions. The main characteristics and nutritional composition of the diets are presented in Table 1. Fatty acid composition of the diets was determined analytically in representative samples from each phase and is presented in Table 1 as the percentage of total identified fatty acids (FAME).

### 2.3. Growth Performance

Growth performance was evaluated in all chickens monitored during the rearing period. Chickens were monitored daily for health status and mortality through routine visual inspection. Individual body weight was recorded weekly from hatching until birds reached the target slaughter weight (SW). Average daily gain (ADG) was calculated individually as the change in body weight over time divided by the number of days between consecutive measurements. Feed intake was recorded at the pen × genotype level throughout the rearing period. Feed conversion ratio (FCR) was calculated for each pen × genotype group as the ratio of total feed supplied to total body weight gain over the complete growing period. Because feed intake was recorded at the group level, FCR was interpreted descriptively and was not subjected to individual-level statistical inference.

### 2.4. Slaughtering Procedure and Carcass Quality Parameters

When chickens reached the target SW, they were processed at the commercial local facilities, following the company’s standard protocol. Stunning was performed using a five-stage multiphase oxygen-carbon dioxide system, with birds exposed to progressively decreasing O_2_ concentrations (24%, 21%, 15%, 0%, 0%) and increasing CO_2_ concentrations (20%, 30%, 39%, 54%, and 73%) for 60 s at each stage. Slaughter was carried out by neck incision, followed by plucking. Evisceration was performed manually by trained UPCT personnel, and abdominal fat was collected as readily separable adipose tissue from the visceral package, excluding mesenteric fat, and therefore represents dissectible abdominal fat. After evisceration, carcasses were chilled for 24 h at 4 °C. Carcasses were then weighed and processed according to Jensen [29]. Head, neck, and feet were removed and weighed individually. The remaining carcass was defined as net carcass weight (NCW). Net carcass yield (NCY), and abdominal fat yield were calculated relative to SW. The breast (skinless and boneless), both leg and wings were removed and weighed individually, and their yields were calculated relative to NCW. The left leg (one leg) was dissected into muscle, bone, skin, and adipose separable fat to determine the meat-to-bone ratio. Thigh meat samples from the left dissected leg (approximately 70 g) were minced, placed in Petri dishes, and stored at −80 °C. Samples were subsequently lyophilized using a freeze dryer (LyoBeta PS, Telstar, Terrassa, Spain) for further chemical analyses. The right breast was vacuum-packed and stored at −80 °C until meat quality analyses.

### 2.5. Meat Quality Parameters

Muscle pH was measured at 45 min and 24 h post-mortem in the left breast and thigh using a portable pH meter (HI-98230, Hanna Instruments, Woonsocket, RI, USA) equipped with an ISFET electrode. At 24 h post-mortem, color parameters lightness (L), redness (a*), and yellowness (b*) were measured according to the CIE 1976 color difference formula [30] using a colorimeter (CR-300, Minolta Camera Co., Osaka, Japan). Measurements were taken on the inner surface of the left breast and on the external surface of the left drumstick muscle after skin removal.

Drip loss of the left breast was determined over a 72 h storage period at 4 °C, with sample weights recorded at 0, 24, and 72 h, while cooking loss was determined on the right breast according to Honikel [31]. Texture profile analysis (TPA) of cooked right breast meat was carried out using a texture analyzer (TA.XTplus, Stable Micro Systems, Godalming, Surrey, UK). A total of 30 samples per genotype were analyzed with a 20 mm diameter cylindrical aluminum probe, following the procedure adapted from U-chupaj et al. [32]. The TPA parameters of hardness (N), fracturability (N), springiness (ratio), cohesiveness (ratio), gumminess (N), and chewiness (N·mm) were derived from the force–time curves using Exponent software (v6.2, Stable Micro Systems, Godalming, Surrey, UK).

Lyophilized thigh meat was used to determine the following parameters: Moisture was determined by oven drying at 105 °C until constant weight (ISO 1442:2023 [33]); lipid content was determined using Soxhlet extraction with petroleum ether (SER 158 automatic extractor, VELP Scientifica, Usmate Velate, Italy); and protein content was determined by total nitrogen analysis using a CHN analyzer (CHN 628, LECO Corporation, St. Joseph, MI, USA) and calculated using a conversion factor of 6.25 [34].

The fatty acid composition of diets and lyophilized thigh meat was determined by gas chromatography–mass spectrometry (GC–MS) after preparation of fatty acid methyl esters (FAME), following the method described by O’Fallon et al. [35] with minor modifications. FAMEs were obtained by transesterification using a methanolic KOH solution, with C13:0 used as an internal standard. Analyses were performed using a gas chromatograph (6890, Agilent Technologies, Santa Clara, CA, USA) coupled to a mass spectrometer (5975, Agilent Technologies) and equipped with a DB-23 capillary column. Fatty acids were identified by comparison with commercial FAME standards (37 Component FAME mix, Supelco, Sigma-Aldrich, St. Louis, MO, USA). Individual fatty acids were expressed as percentages of total identified fatty acids. From these data, sums of saturated (SFA), monounsaturated (MUFA), polyunsaturated (PUFA), unsaturated (UFA = MUFA + PUFA), and n-3 and n-6 fatty acids were calculated, as well the ratios UFA/SFA, PUFA/SFA and n-6/n-3.

### 2.6. Statistical Analysis

Statistical analyses were performed using R software (v 4.3.3). The individual animal was considered the experimental unit for all traits, except for FCR and mortality, which were analyzed at the pen level (two per genotype).

Data were analyzed using a general linear model (GLM) including genotype (G; Mur, MurHb, Hb). For carcass traits and chemical composition of thigh meat, SW was included as a covariate. For cooking loss, initial breast portion weight was included as a covariate, whereas FCR and mortality data were not subjected to inferential statistical analysis and were presented descriptively. Homogeneity of variances and normality of residuals were assessed using Levene’s and Kolmogorov–Smirnov tests, respectively.

Data are reported as least square means ± standard error (SE). Pairwise comparisons among genotypes were performed using Bonferroni adjustment for multiple testing. Statistical significance was set at *p* < 0.05. FCR is reported as mean and standard deviation (SD).

In addition, an exploratory Principal Component Analysis (PCA) was performed to summarize the multivariate structure of breast meat quality traits. The analysis included ultimate pH, color parameters (L*, a* and b*), drip loss at 24 h of storage, cooking loss, and instrumental texture traits. Variables were centered and scaled to unit variance before analysis. Component interpretation was based on eigenvalues, explained variance, and variable loadings. Individual scores were represented on the first two principal components, together with 95% confidence ellipses for genotype groups, and the corresponding loading plot was used to identify the main traits contributing to genotype differentiation. Python (v.3) was used for graphical representation.

## 3. Results

### 3.1. Growth Performance

Growth performance parameters are presented in Table 2. Initial body weight differed among genotypes, being highest in Mur, intermediate in MurHb, and lowest in Hb. In contrast, ADG showed the opposite pattern, with the lowest values observed in Mur, intermediate values in MurHb, and the highest values in Hb. Consequently, Mur birds reached the slaughter target weight at older ages (126 days), followed by MurHb (78 days) and finally Hb birds (53 days).

The difference in growth rate between the crossbred MurHb birds and the Hb genotype was considerably greater than that observed between MurHb and Mur chickens. In this regard, the difference in ADG between MurHb and Hb represented 67.7% of the overall mean value, whereas the difference between MurHb and Mur accounted for 25.6%.

Although no significant differences in FCR could be confirmed due to the limited number of observations, a descriptive trend was observed, with FCR decreasing progressively from Mur to MurHb and Hb.

These results were not influenced by health-related problems. Throughout the experimental period, two Mur and two Hb chickens died, corresponding to mortality rates of 3.8% and 3.7%, respectively.

### 3.2. Carcass Traits and Meat Quality

Results for slaughter performance and carcass characteristics are presented in Table 3. The NCW was higher in MurHb than in Mur (*p* < 0.05), although lower than in Hb, and the same pattern was observed for NCY, with the MurHb chickens showing values that were practically intermediate between the two parental genotypes. Breast and legs weight and yield, and wing yield were markedly influenced by genotype (*p* < 0.001). The most pronounced difference was observed in breast yield, Hb showed the highest value, followed by MurHb and finally Mur, whereas the opposite pattern was observed for the yield of the two legs. For both traits, the values observed in the MurHb birds were closer to those of Mur than to those of Hb. Thus, MurHb showed values that were 11.7% higher than Mur and 38.6% lower than Hb, relative to an overall mean breast yield of 22.3%. Regarding leg cuts, Mur and MurHb showed similar thigh and drumstick percentages, as well as a similar thigh meat-to-bone ratio, whereas Hb birds exhibited the lowest drumstick percentage but the highest thigh percentage and thigh meat-to-bone ratios. No genotype differences were observed in drumstick meat-bone ratio. About abdominal fat yield, Mur and MurHb exhibited higher values than Hb. However, MurHb showed significant differences regarding thigh separable fat percentage than Hb and comparable values with Mur.

Regarding meat quality (Table 4), breast pH measured at 45 min post-mortem was highest in Mur birds, whereas in the thigh, both Mur and MurHb showed higher pH values than Hb birds. In the breast, Mur showed higher pH values at 24 h post-mortem than Hb birds and those higher than MurHb. In the thigh, MurHb birds presented lower pH values than Hb birds. Notably, Hb exhibited a slower decline in thigh pH at 24 h post-mortem. Breast and thigh meat color parameters were significantly influenced by genotype (*p* < 0.001), but the pattern was different. The breast and thigh L* was lower in Mur and MurHb than Hb, with MurHb resembling Hb. For a*, MurHb chickens showed lower values in the breast than both Mur and Hb, which exhibited similar values. In the thigh, Mur birds presented the highest values, while MurHb and Hb chickens showed comparable values. For b*, Mur birds showed the lowest values in both breast and thigh muscle, followed by MurHb and Hb, while MurHb exhibited intermediate values in thigh. About drip loss, at 24 h of storage, MurHb and Hb chickens exhibited higher drip loss than Mur birds, with no significant differences between MurHb and Hb. Drip loss further increased at 72 h of storage, particularly in Hb birds, resulting in significant differences among all genotypes. Mur birds showed the lowest drip loss values, MurHb displayed intermediate values, and Hb birds exhibited the highest values. A similar pattern was observed for cooking loss, which increased progressively from Mur to MurHb and Hb, although the crossbred group showed values closer to Mur than to Hb. About TPA profile, MurHb and Mur showed similar hardness, gumminess and chewiness values, whereas Hb exhibited higher values for these parameters. Springiness was significantly higher in MurHb than Mur and Hb. Cohesiveness showed minor differences among genotypes, with Mur displaying slightly higher values than Hb (*p* < 0.05).

Chemical composition was measured for thigh meat and is presented in Table 4. An effect of genotype (*p* < 0.001) on both protein and fat percentages was revealed, which, as expected, followed opposite patterns. The most pronounced differences were observed for fat content. In this regard, lipid content increased progressively from Mur to MurHb and Hb, with differences of 0.66% and 0.70% between consecutive genetic groups, respectively. These differences represented 12.1% and 12.9% of the overall mean fat content (5.43%). In contrast, protein percentage followed the opposite trend, with the highest values observed in Mur, followed by MurHb and finally Hb. In this case, differences were smaller, amounting to 0.9% between Mur and MurHb and 0.7% between MurHb and Hb, representing 4.7% and 2.2% of the overall mean protein content (19.2%), respectively.

### 3.3. Principal Component Analysis

**Figure 2:**
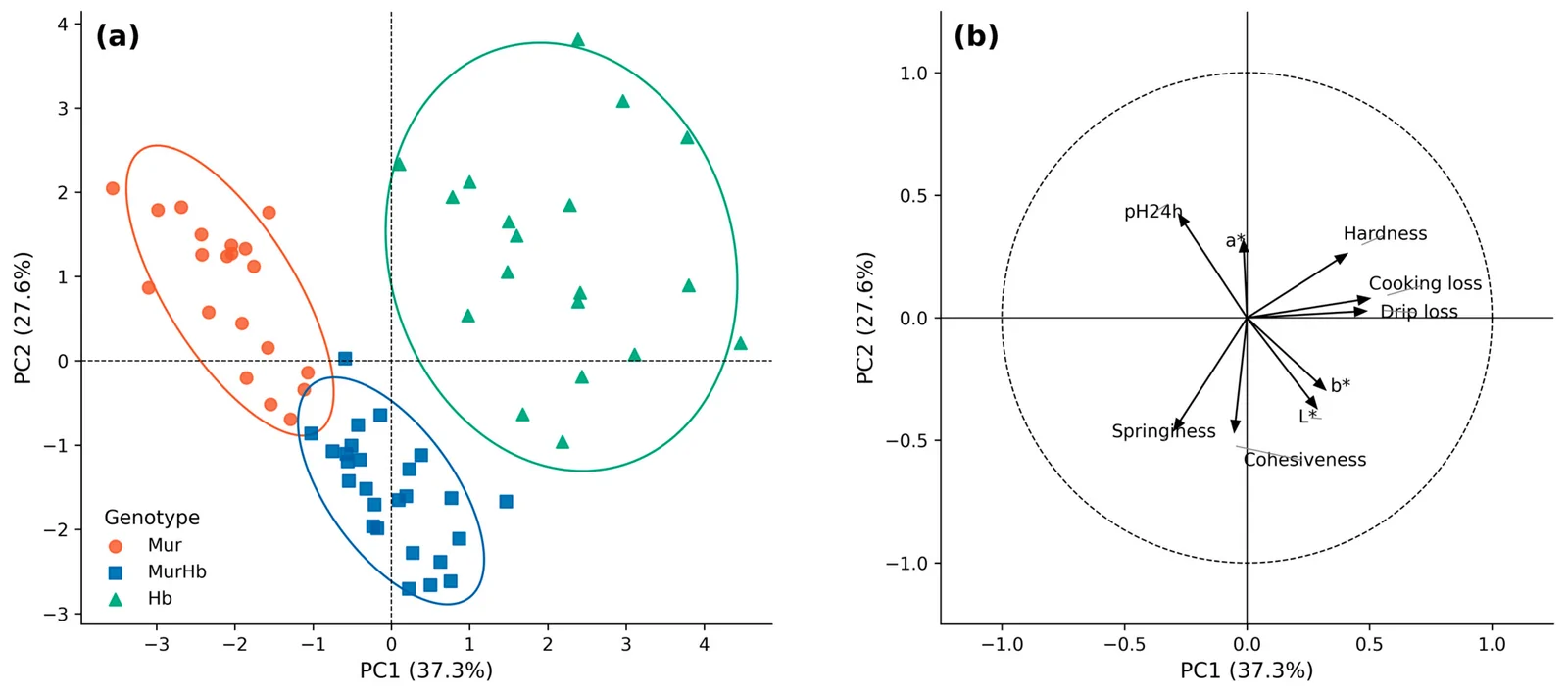
Principal Component Analysis (PCA) of breast meat quality traits. L*: lightness; a*: redness; b*: yellowness; drip loss at 24 h of storage. (**a**) Score plot of individual chicken from the Murciana breed (Mur), the commercial hybrid Hubbard JA757 (Hb), and their F1 crossbred group (MurHb) on the first two principal components. (**b**) Loading plot shows the contribution of breast meat quality traits to PC1 and PC2.

Figure 2 shows the PCA performed on breast meat quality traits. The first two principal components explained 64.9% of the total variance, while the first three components accounted for 77.8% (Table 5). In the PC1–PC2 score plot, the three genotypes showed a structured distribution (Figure 2a). Mur and Hb were mainly separated along PC1, with Mur distributed towards negative PC1 values and Hb towards positive PC1 values. The MurHb group occupied an intermediate position along PC1, although it was displaced towards negative PC2 values, partially separating from both parental genotypes.

The loading plot indicated that PC1 was mainly associated with cooking loss, drip loss and hardness on the positive side, and with springiness and pH24 on the negative side (Figure 2b; Table 6). PC2 was mainly related to pH24 on the positive side and to springiness, cohesiveness and L* on the negative side. PC3, although not represented in Figure 2, was primarily associated with chromatic traits, particularly a* and b* (Table 6).

### 3.4. Fatty Acid Profile

The fatty acid composition is presented in Table 7 and was characterized by proportions of 33–35% of SFA, 36–42% of MUFA, and 25–30% of PUFA. Total SFA content was higher in Mur birds than in MurHb and Hb birds. Among SFA, palmitic acid (C16:0) was the most abundant fatty acid, accounting for approximately 22.4–23.7% of total fatty acids, being slightly lower in Mur than in Hb. Stearic acid (C18:0) was the second most abundant SFA, with an average proportion of 9.66%, and was present at higher levels in Mur than in MurHb and those higher than in Hb birds. For several minor SFA, higher levels of C14:0, C15:0, C17:0, C20:0, and C22:0 were observed in Mur than in Hb chickens and, in the end, the greater SFA proportion was observed in Mur birds compared with Hb birds. MurHb birds showed intermediate SFA values between Mur and Hb for most individual fatty acids, except for C14:0, for which MurHb and Mur birds did not differ significantly. However, total SFA content of MurHb birds was similar to that of Hb birds. In contrast, total MUFA content showed the opposite trend, increasing progressively from Mur to MurHb and Hb. This pattern was mainly driven by oleic acid (C18:1 n-9), the predominant MUFA, with an overall mean value of 34.5%, for which Hb birds presented the highest values. The second most abundant MUFA was palmitoleic acid (C16:1 n-7), although its proportion was considerably lower (overall mean 3.28%). Its highest levels were observed in Hb birds, followed by MurHb and finally Mur birds. MurHb birds resembled Mur for most MUFA, including C17:1, C18:1, C20:1 and total MUFA, except for C14:1, in which MurHb and Hb did not differ significantly. Total PUFA content was also significantly affected by genotype (*p* < 0.001) and was higher in Mur and MurHb than in Hb birds. The differences were observed for specific PUFA C18:2 n-6, C18:3 n-6, C20:3 n-6, C20:4 n-6 and C18:3 n-3 (*p* < 0.05). Among n-6 fatty acids, linoleic acid (C18:2 n-6; LA) represented the most abundant PUFA (overall mean 19.2%) and was higher in Mur and MurHb than in Hb chickens. Among n-3 fatty acids, alpha-linolenic acid (C18:3 n-3; ALA) was the most representative acid and the second higher PUFA and was higher in Mur and MurHb than in Hb. Eicosapentaenoic acid (C20:5 n-3, EPA) was also detected, albeit at low concentrations, with no significant effect of genotype observed. The n-6/n-3 ratio was lower in Mur and MurHb than in Hb birds, although no significant differences were observed between Mur and MurHb. Regarding the UFA/SFA ratio, Hb birds showed higher values than Mur and MurHb birds. In contrast, the PUFA/SFA ratio showed the opposite pattern, and Mur and MurHb showed comparable values and were higher than in Hb birds (*p* < 0.05).

## 4. Discussion

Crossbreeding has been proposed as a practical strategy to improve the productive performance of local chicken breeds while retaining part of their genetic, phenotypic, and product-related identity, thereby supporting the functional use of local genetic resources within conservation-oriented production systems [7,8,10,14]. Under the common target slaughter-weight criterion used in the present study, MurHb chickens showed a clear improvement in productive performance relative to the local breed, while carcass and meat-quality traits shifted only partially towards those observed in the commercial genotype. This response is central to the interpretation of the study: MurHb chickens did not behave as a simple intermediate genotype for all traits, nor did they reproduce the biological profile of Hb. Instead, the crossbred group expressed a trait-specific phenotype, with marked gains in growth rate and carcass yield, but with several structural, textural, and lipid-related attributes remaining closer to Mur than to Hb.

Growth performance was the trait group most clearly improved by crossbreeding. At a common target slaughter weight, MurHb birds reached slaughter weight approximately 42 days earlier than Mur birds and showed higher ADG, together with a marked reduction in FCR. However, their performance remained clearly below that of Hb, showing that MurHb achieved a substantial improvement in productive efficiency relative to Mur without reaching the commercial-type growth pattern of Hb. Comparable responses have been reported for previous local × commercial crosses. Cassandro et al. [17] observed improved productive and carcass traits in Padovana and commercial crosses compared with the local breed, although without fully reproducing the commercial profile, while Fiorilla et al. [7] similarly observed improved productive performance in crosses between Italian local breeds and a commercial strain under both conventional and free-range systems. Dzungwe et al. [14] reported intermediate productive responses in reciprocal crosses between Sasso and Wassachie chickens, with the magnitude of improvement depending on the direction of the cross. Overall, these studies indicate that productive responses to crossbreeding depends strongly on the parental genotypes, cross direction, and production system [16,19,20].

The carcass results further support this interpretation. MurHb showed higher carcass weight and yield than Mur, but lower values than Hb, indicating improved slaughter performance while maintaining an intermediate position between parental genotypes. However, the distribution of commercial cuts did not shift uniformly towards Hb. Breast yield increased in MurHb compared with Mur, which represents an important productive advantage because breasts are the most economically valuable cut in broiler production. Nevertheless, MurHb remained much closer to Mur than to Hb for breast yield and also retained a higher relative proportion of leg quarters and wings than Hb. This pattern is consistent with the marked contrast between commercial meat-hybrid chickens, which have been intensively selected for pectoral muscle development, and local or slow-growing genotypes, which often show a less breast-oriented carcass conformation and relatively greater development of other cuts [36,37]. Together with the similar leg quarter meat-to-bone ratio observed in Mur and MurHb, these results indicate that MurHb improved carcass efficiency relative to Mur, but did not fully redefine carcass conformation.

Meat-quality traits showed a more complex and muscle-dependent response than growth and carcass traits. In breast meat, MurHb showed lower ultimate pH than both parental genotypes, whereas in thigh meat the post-mortem pattern also differed among genotypes, again with MurHb showing the lowest ultimate pH. Such muscle-specific responses are expected because breast and thigh muscles differ in fiber type, oxidative metabolism, pigment content, and post-mortem glycolytic dynamics. Genotype and production background have been repeatedly identified as major factors affecting poultry meat pH, color, water-holding capacity, and texture, particularly when birds with different growth rates are compared [36,38,39,40,41]. Therefore, the present results indicate a trait- and muscle-specific meat quality response in MurHb rather a uniform intermediate phenotype. Color traits reinforced this interpretation. MurHb resembled Mur in breast lightness, but resembled Hb in thigh lightness, while redness was markedly reduced in both muscles compared with Mur. In contrast, yellowness increased from Mur to Hb, with MurHb generally showing an intermediate or closer-to-Hb response. Meat color in poultry is influenced by genotype, muscle type, pigment concentration, fiber metabolism, age, diet, and rearing conditions, and previous comparisons among local and commercial genotypes have reported substantial variability in L*, a*, and b* values depending on the muscle evaluated and the production background [37,41,42]. Thus, the observed color response suggests that crossbreeding modified the visual profile of Murciana meat, but not through a generalized shift towards Hb. Instead, MurHb showed a muscle-dependent color phenotype, reinforcing the idea that the F1 response cannot be reduced to a simple midpoint between parental genotypes.

Breast meat quality was also differed among genotypes. Mur birds showed the lowest drip and cooking losses, whereas Hb birds showed the highest values, and MurHb generally occupied an intermediate position. This indicates that crossbreeding reduced the water-holding capacity of Mur meat, although the crossbred birds did not reach the poorer water-retention profile observed in Hb, particularly for 72 h drip loss and cooking loss. A lower ultimate pH is commonly associated with reduced water-holding capacity and increased drip or cooking losses because post-mortem acidification affects protein denaturation and the ability of muscle tissue to retain water [31,38,42]. Therefore, the lower ultimate pH observed in MurHb breast meat may partly explain its higher water losses compared with Mur. Similar intermediate or trait-specific responses have also been described in local × commercial chicken crosses [14,17,19]. Texture traits followed a different pattern. MurHb did not differ from Mur in hardness, gumminess, or chewiness, whereas Hb showed higher values for these parameters. This suggests that, despite the changes observed in pH, color, and water losses, the crossbred birds maintained a texture profile closer to the local breed than to the commercial line. Previous studies have reported variable relationships between genotype, growth rate, and meat texture, with responses depending on factors such as slaughter age, muscle characteristics, post-mortem metabolism, and processing conditions [36,43]. Because texture analysis was performed after frozen storage, the potential influence of freezing–thawing and deboning time procedures on instrumental texture should also be considered [44,45]. The meat quality trait-specific interpretation was also supported by the multivariate analysis of meat-quality traits. The PCA did not show a complete overlap of MurHb with either parental genotype but rather positioned the crossbred group between Mur and Hb, with partial proximity to Mur for several technological and textural attributes. This multivariate pattern reinforces the univariate results, indicating that MurHb modified the overall meat-quality profile of Murciana chickens without fully shifting it towards the commercial genotype.

The chemical composition of thigh meat also reflected a partial shift from Mur towards Hb. Moisture did not differ among genotypes, but protein and lipid content followed opposite patterns. Mur showed the highest protein and lowest lipid percentages, Hb showed the lowest protein and highest lipid percentages, and MurHb occupied an intermediate position, although closer to Hb for protein and intermediate for lipid deposition. Genotype-related differences in meat composition have been widely reported in chickens, with slow-growing and local genotypes often differing from commercial lines in muscle development, fat deposition, and protein content [6,36,46,47].

The fatty acid profile of thigh meat was shaped by the combined influence of the n-3-enriched feeding program and differences among genotypes. The exceptionally high ALA content of the finisher diet was likely the main driver of the elevated n-3 PUFA levels observed across all three genotypes, consistent with the well-established close relationship between dietary fatty acid supply and tissue deposition in poultry [48]. Nevertheless, significant differences among genotypes were observed for most individual and grouped fatty acids. Total SFA was lower in MurHb than in Mur and reached values similar to Hb, mainly due to the marked reduction in stearic acid (C18:0). Conversely, total MUFA was highest in Hb, largely driven by its higher oleic acid (C18:1 n-9) content, whereas Mur and MurHb did not differ significantly for this fraction. In contrast, MurHb maintained total PUFA, n-6, n-3, n-6/n-3 ratio, and PUFA/SFA values comparable to Mur and clearly distinct from Hb. The higher ALA proportions observed in Mur and MurHb compared with Hb, despite the common diet, may reflect genotype-related differences in dietary fatty acid deposition or retention, possibly associated with differences in lipid metabolism [49,50]. EPA was detected only in minor proportions and did not differ significantly among genotypes, which is consistent with the limited effectiveness of ALA as a precursor of EPA deposition in edible poultry tissues [48]. The n-6/n-3 ratio further reflected the strong effect of the ALA-enriched diet. Mur and MurHb showed values below the 4:1 ratio often used as a nutritional reference [51], whereas Hb was only slightly above this threshold. These ratios were markedly lower than those commonly reported in poultry meat from autochthonous breeds or conventional feeding conditions, where values frequently within the 10:1–20:1 range have been described [37,39,52]. However, under this common dietary background, MurHb did not simply shift towards the lipid profile of Hb but combined reduced SFA relative to Mur with the preservation of MUFA- and PUFA-related traits closer to the local breed.

From a production-system perspective, the findings illustrate that substantial improvements in efficiency can be achieved without fully abandoning the phenotypic identity of the local breed. MurHb does not approach the performance ceiling of specialized broilers, nor does it replicate the slow-growth profile of Mur. Instead, it represents a compromise genotype combining improved productivity with retention of key structural and quality-related characteristics. This type of genotype may be particularly relevant for differentiated or alternative production systems where moderate growth, product identity, local origin, and consumer perception of quality or welfare are valued [12,53,54]. Within conservation frameworks, this balance is particularly relevant given the progressive erosion of local poultry genetic resources in Europe [4,55,56] and the need for economically viable utilization strategies that provide incentives for farmers and maintain the functional relevance of local breeds [8,9,10,57]. In this context, crossbreeding should not be viewed as a substitute for purebred conservation, but as a complementary tool within conservation-through-use schemes, capable of enhancing the productive valorization of Murciana-derived animals under specific market conditions.

Some aspects of the experimental design should be considered when interpreting these results and may guide future research. Birds were deliberately slaughtered at a common live weight instead of at a common age, which allowed carcass and meat-quality traits to be compared at a similar market weight, but also implied differences in physiological maturity among genotypes. Because carcass composition, muscle metabolism, and meat-quality traits may vary with age in poultry, the observed differences should be interpreted in the context of these divergent growth trajectories [24,39,47,58]. Future studies could further account for these differences by considering the degree of physiological maturity of each genotype, for example through genotype-specific growth curves. In addition, the feeding program was based on the commercial Hb schedule and then applied to Mur and MurHb, meaning that the local and crossbred genotypes may not have been evaluated under diets specifically optimized for their growth rate or nutrient requirements. Genotype × diet interactions may influence growth, carcass yield, and meat-quality traits in chickens with different growth rates [25,36]. Finally, only one cross direction was evaluated, using Mur males and Hb females, and only male birds were included because females were retained for breeding within the conservation program. Future studies including reciprocal crosses and female birds would help clarify the potential role of maternal effects, sex-linked inheritance, cross direction, and sex on the productive and meat-quality performance of Murciana-derived crossbreds [14,25,43,59].

## 5. Conclusions

The present study shows that crossing Murciana chickens with a medium-growing commercial genotype substantially improves productive performance and carcass yield relative to the local breed, without fully shifting the crossbred birds towards the commercial biological profile. MurHb expressed a trait-specific response, combining higher growth efficiency with the retention of several carcass, textural, and lipid-related attributes closer to Mur than to Hb.

These findings support the potential use of Murciana-derived crossbreds as a complementary strategy to improve the productive valorization of the breed within differentiated or alternative poultry systems. However, crossbreeding should not be regarded as a substitute for purebred conservation, but rather as an additional tool within conservation-through-use frameworks. Further research should assess the stability and practical value of this strategy under different crossing schemes, dietary programs, and market-oriented production conditions.

## Figures and Tables

**Table 1:** Composition and nutritional characteristics of the feeding program across growth phases.

Parameter	Starter	Early Grower	Late Grower	Finisher
Metabolizable Energy (kcal/kg)	2850–2900	2950–3000	2950–3000	3000–3050
Crude protein (%)	21.7	19.8	18.3	16.7
Crude fat (%)	4.8	5.2	5.9	5.0
Crude fiber (%)	2.7	2.7	2.6	2.4
Starch (%)	39.4	43.9	45.1	47.6
Sugars (%)	4	3.6	3.4	3.1
Ash (%)	6.1	5.1	4.4	3.6
Calcium (%)	0.9	0.8	0.7	0.6
Total Phosphorus (%)	0.6	0.5	0.4	0.3
*Major fatty acids (% of total identified fatty acids)*				
C16:0	17.4	17.4	15.83	12.23
C18:0	4.27	4.02	3.08	3.73
C18:1 n-9	25.0	26.4	28.2	25.92
C18:2 n-6	47.5	46.2	47.9	31.49
C18:3 n-3	4.07	4.29	3.08	25.14
SFA	23.0	22.6	20.2	17.00
MUFA	25.5	26.9	28.8	26.37
PUFA	51.6	50.5	51.0	56.63
PUFA/SFA	2.24	2.23	2.52	3.36
n-6/n-3	11.7	10.8	16.57	1.26

Fatty acid composition of the diet expressed as percentage of total identified fatty acids. SFA: saturated fatty acids; MUFA: monounsaturated fatty acids; PUFA: polyunsaturated fatty acids; n-6/n-3: ratio between n-6 and n-3 fatty acids.

**Table 2:** Growth performance of Murciana chicken (Mur), a commercial hybrid (Hb), and their crossbred group (MurHb).

Parameter	Mur (*n* = 54)	MurHb (*n* = 54)	Hb (*n* = 66)	G (*p*-Value)
Initial body weight (g)	42.9 ^a^ ± 0.55	40.5 ^b^ ± 0.48	37.7 ^c^ ± 0.40	<0.001
Final body weight (g)	2726 ^b^ ± 34.5	2763 ^b^ ± 35.4	2900 ^a^ ± 30.1	0.001
ADG (g/d)	21.5 ^c^ ± 0.49	30.6 ^b^ ± 0.50	54.7 ^a^ ± 0.43	<0.001
FCR	3.89 ± 0.31	2.58 ± 0.30	1.93 ± 0.11	–

Data are presented as least square means ± standard error (SE), except for age at slaughter and FCR which is expressed as mean ± standard deviation (SD). ADG: average daily gain; FCR: feed conversion ratio; Mur: Murciana; MurHb: Murciana × Hubbard JA757; Hb: Hubbard JA757; G: genetic type; –: not applicable, as the genotype effect was not statistically tested for FCR. Different superscript letters (^a, b, c^) within a row indicate significant differences among genotypes (*p* < 0.05).

**Table 3:** Carcass and cut traits of Murciana chicken (Mur), a commercial hybrid (Hb), and their crossbred group (MurHb).

	Mur (*n* = 52)	MurHb (*n* = 54)	Hb (*n* = 64)	G (*p*-Value)
*Carcass and cut weights*				
SW (g)	2722 ^b^ ± 36.4	2763 ^b^ ± 35.5	2891 ^a^ ± 30.2	0.001
NCW (g)	1700 ^c^ ± 14.2	1805 ^b^ ± 13.8	1927 ^a^ ± 12.2	<0.001
Breast (g)	302 ^c^ ± 6.73	366 ^b^ ± 6.51	563 ^a^ ± 5.78	<0.001
Two legs (g)	617 ^b^ ± 4.93	635 ^a^ ± 4.77	597 ^c^ ± 4.23	<0.001
Abdominal fat (g)	36.9 ^a^ ± 2.57	36.7 ^a^ ± 2.49	28.5 ^b^ ± 2.21	0.017
*Carcass and cut yields*				
NCY ^1^ (%)	61.3 ^c^ ± 0.23	65.1 ^b^ ± 0.22	68.2 ^a^ ± 0.20	<0.001
Wings ^2^ (%)	12.6 ^a^ ± 0.11	12.5 ^a^ ± 0.11	10.6 ^b^ ± 0.09	<0.001
Breast ^2^ (%)	17.7 ^c^ ± 0.23	20.3 ^b^ ± 0.22	28.9 ^a^ ± 0.19	<0.001
Two legs ^2^ (%)	36.4 ^a^ ± 0.17	35.2 ^b^ ± 0.17	31.1 ^c^ ± 0.15	<0.001
Abdominal fat yield ^1^ (%)	1.3 ± 0.09	1.3 ± 0.09	1.0 ± 0.08	0.022
*Leg dissection*				
Thigh ^3^ (%)	53.2 ^b^ ± 0.63	52.4 ^b^ ± 0.61	56.3 ^a^ ± 0.54	<0.001
Drumstick ^3^ (%)	47.1 ^a^ ± 0.57	46.5 ^a^ ± 0.56	43.5 ^b^ ± 0.48	<0.001
Thigh meat-to-bone ratio ^3^	2.7 ^b^ ± 0.12	3.1 ^b^ ± 0.12	4.3 ^a^ ± 0.11	<0.001
Drumstick meat-to-bone ratio ^3^	2.4 ± 0.05	2.4 ± 0.05	2.5 ± 0.04	0.190
Thigh separable fat ^3^ (%)	1.2 ^ab^ ± 0.09	1.0 ^b^ ± 0.08	1.3 ^a^ ± 0.07	0.034

Data are presented as least square means ± standard error (SE). SW: slaughter weight; NCW: net carcass weight; NCY: net carcass yield; Mur: Murciana; MurHb: Murciana × Hubbard JA757; Hb: Hubbard JA757; G: genotype. ^1^ yield expressed relative to SW; ^2^ yields expressed relative to NCW; ^3^ yields expressed relative to one leg. All traits, except SW, were adjusted to the mean SW of 2776.39 g. Different superscript letters (^a, b, c^) within a row indicate significant differences among genotypes (*p* < 0.05).

**Table 4:** Meat quality parameters of breast and thigh meat, of the breast of Murciana chicken (Mur), a commercial hybrid (Hb), and their crossbred group (MurHb).

	Mur (*n* = 52)	MurHb (*n* = 54)	Hb (*n* = 64)	G (*p*-Value)
*Breast*				
pH 45 min	6.57 ^a^ ± 0.03	6.32 ^b^ ± 0.03	6.40 ^b^ ± 0.02	<0.001
pH 24 h	5.98 ^a^ ± 0.02	5.75 ^c^ ± 0.02	5.86 ^b^ ± 0.02	<0.001
pH decline	0.58 ^a^ ± 0.03	0.58 ^a^ ± 0.03	0.46 ^b^ ± 0.03	0.018
L*	40.5 ^b^ ± 0.25	41.8 ^a^ ± 0.38	41.7 ^a^ ± 0.22	<0.001
a*	1.39 ^a^ ± 0.08	0.85 ^b^ ± 0.12	1.40 ^a^ ± 0.07	<0.001
b*	7.13 ^b^ ± 0.20	8.84 ^a^ ± 0.30	9.23 ^a^ ± 0.17	<0.001
Drip loss 24 h (%)	0.50 ^b^ ± 0.04	0.75 ^a^ ± 0.04	0.85 ^a^ ± 0.03	<0.001
Drip loss 72 h (%)	0.85 ^c^ ± 0.05	1.13 ^b^ ± 0.06	1.47 ^a^ ± 0.04	<0.001
Cooking loss ^1^ (%)	11.8 ^c^ ± 0.59	13.7 ^b^ ± 0.46	20.1 ^a^ ± 0.79	<0.001
Hardness (N)	5.57 ^b^ ± 0.30	5.63 ^b^ ± 0.30	8.83 ^a^ ± 0.30	<0.001
Springiness (ratio)	0.76 ^b^ ± 0.008	0.82 ^a^ ± 0.008	0.70 ^c^ ± 0.008	<0.001
Cohesiveness (ratio)	0.62 ^ab^ ± 0.004	0.63 ^a^ ± 0.004	0.61 ^b^ ± 0.004	0.034
Gumminess (N)	3.43 ^b^ ± 0.19	3.56 ^b^ ± 0.19	5.39 ^a^ ± 0.19	<0.001
Chewiness (N⋅mm)	2.61 ^b^ ± 0.14	2.93 ^b^ ± 0.14	3.77 ^a^ ± 0.14	<0.001
*Thigh*				
pH 45 min	6.34 ^a^ ± 0.03	6.34 ^a^ ± 0.03	6.23 ^b^ ± 0.02	0.006
pH 24 h	6.14 ^ab^ ± 0.02	6.06 ^b^ ± 0.02	6.20 ^a^ ± 0.02	<0.001
pH decline	0.24 ^a^ ± 0.02	0.26 ^a^ ± 0.03	0.04 ^b^ ± 0.03	<0.001
L*	40.3 ^b^ ± 0.25	41.7 ^a^ ± 0.37	41.3 ^a^ ± 0.21	0.001
a*	3.39 ^a^ ± 0.13	1.69 ^b^ ± 0.20	1.85 ^b^ ± 0.11	<0.001
b*	5.71 ^c^ ± 0.21	7.24 ^b^ ± 0.32	8.58 ^a^ ± 0.18	<0.001
Moisture ^2^ (%)	72.6 ± 0.15	72.7 ± 0.22	72.8 ± 0.13	0.678
Protein ^2^ (%)	19.9 ^a^ ± 0.13	19.0 ^b^ ± 0.19	18.6 ^b^ ± 0.11	<0.001
Lipids ^2^ (%)	4.76 ^c^ ± 0.15	5.42 ^b^ ± 0.23	6.12 ^a^ ± 0.13	<0.001

Data are presented as least square means ± standard error (SE). pH at 45 min and 24 h post-mortem; L*: lightness; a*: redness; b*: yellowness; drip loss at 24 h and 72 h of storage, respectively; Mur: Murciana; MurHb: Murciana × Hubbard JA757; Hb: Hubbard JA757; G: genotype. ^1^ Adjusted for the mean initial breast portion weight of 144.27 g. ^2^ Adjusted for the mean SW of 2776.39 g. For texture profile analysis, *n* = 30 per genotype. Different superscript letters (^a, b, c^) within a row indicate significant differences among genotypes (*p* < 0.05).

**Table 5:** Variance explained by the first principal components of the PCA performed on breast meat quality traits.

Component	Eigenvalue	% Variance	Cumulative %
PC1	3.355	37.3	37.3
PC2	2.487	27.6	64.9
PC3	1.160	12.9	77.8

**Table 6:** Loadings of breast meat quality traits on the first three principal components.

Variable	PC1	PC2	PC3
pH 24 h	−0.278	0.421	0.300
L*	0.284	−0.368	0.337
a*	−0.015	0.315	−0.697
b*	0.319	−0.293	−0.508
Drip loss	0.485	0.028	0.022
Cooking loss	0.499	0.079	0.054
Hardness	0.407	0.261	0.199
Springiness	−0.298	−0.459	−0.082

L*: lightness; a*: redness; b*: yellowness; drip loss at 24 h of storage.

**Table 7:** Fatty acid profile of thigh (% of total identified fatty acids) of Murciana chicken (Mur), a commercial hybrid (Hb), and their crossbred group (MurHb).

Fatty Acid	Mur (*n* = 52)	MurHb (*n* = 54)	Hb *(n* = 64)	G (*p*-Value)
C14:0	0.55 ^a^ ± 0.01	0.57 ^a^ ± 0.01	0.50 ^b^ ± 0.01	<0.001
C15:0	0.12 ^a^ ± 0.003	0.10 ^b^ ± 0.002	0.08 ^c^ ± 0.002	<0.001
C16:0	22.4 ^b^ ± 0.29	23.3 ^ab^ ± 0.25	23.7 ^a^ ± 0.21	0.003
C17:0	0.27 ^a^ ± 0.01	0.18 ^b^ ± 0.01	0.13 ^c^ ± 0.01	<0.001
C18:0	11.61 ^a^ ± 0.18	9.21 ^b^ ± 0.16	8.17 ^c^ ± 0.13	<0.001
C20:0	0.21 ^a^ ± 0.01	0.14 ^b^ ± 0.01	0.10 ^c^ ± 0.01	<0.001
C22:0	0.08 ^a^ ± 0.01	0.05 ^b^ ± 0.01	0.00 ^c^	<0.001
∑SFA	35.1 ^a^ ± 0.44	33.5 ^b^ ± 0.37	32.5 ^b^ ± 0.32	<0.001
C14:1 n-5	0.06 ^b^ ± 0.004	0.10 ^a^ ± 0.003	0.11 ^a^ ± 0.003	<0.001
C16:1 n-7	2.00 ^c^ ± 0.13	3.48 ^b^ ± 0.12	4.36 ^a^ ± 0.10	<0.001
C17:1 n-7	0.14 ^a^ ± 0.01	0.12 ^ab^ ± 0.01	0.11 ^b^ ± 0.01	<0.001
C18:1 n-9	33.2 ^b^ ± 0.45	33.0 ^b^ ± 0.38	37.4 ^a^ ± 0.33	<0.001
C20:1 n-9	0.39 ^ab^ ± 0.01	0.37 ^b^ ± 0.01	0.41 ^a^ ± 0.01	0.003
∑MUFA	35.7 ^b^ ± 0.49	36.8 ^b^ ± 0.42	42.2 ^a^ ± 0.36	<0.001
C18:2 n-6	20.3 ^a^ ± 0.42	20.8 a ± 0.36	18.6 ^b^ ± 0.30	<0.001
C18:3 n-6	0.18 ^b^ ± 0.01	0.21 ^a^ ± 0.01	0.18 ^b^ ± 0.01	<0.001
C20:2 n-6	0.25 ± 0.01	0.26 ± 0.01	0.25 ± 0.01	0.567
C20:3 n-6	0.19 ^b^ ± 0.01	0.25 ^a^ ± 0.01	0.27 ^a^ ± 0.01	<0.001
C20:4 n-6	1.57 ^a^ ± 0.05	1.46 ^a^ ± 0.04	1.23 ^b^ ± 0.04	<0.001
C18:3 n-3	7.00 ^a^ ± 0.26	6.62 ^a^ ± 0.22	4.99 ^b^ ± 0.19	<0.001
C20:5 n-3	0.33 ± 0.03	0.29 ± 0.02	0.24 ± 0.03	0.077
∑PUFA	29.3 ^a^ ± 0.67	29.6 ^a^ ± 0.58	25.2 ^b^ ± 0.49	<0.001
n6	22.2 ^a^ ± 0.45	22.8 ^a^ ± 0.39	20.2 ^b^ ± 0.33	<0.001
n3	7.11 ^a^ ± 0.27	6.87 ^a^ ± 0.23	5.04 ^b^ ± 0.20	<0.001
n-6/n-3	3.30 ^b^ ± 0.13	3.45 ^b^ ± 0.11	4.12 ^a^ ± 0.09	<0.001
UFA/SFA	1.89 ^b^ ± 0.03	1.99 ^b^ ± 0.03	2.08 ^a^ ± 0.02	<0.001
PUFA/SFA	0.87 ^a^ ± 0.03	0.89 ^a^ ± 0.02	0.78 ^b^ ± 0.02	<0.001

Data are presented as least square means ± standard error (SE). SFA: saturated fatty acids; MUFA: monounsaturated fatty acids; PUFA: polyunsaturated fatty acids; n-6/n-3: ratio between n-6 and n-3 fatty acids; UFA: unsaturated fatty acids; G: genotype. Different superscript letters (^a, b, c^) within a row indicate significant differences among genotypes (*p* < 0.05).

## Data Availability

The relevant data are included within the article. Extra-data can be obtained from the corresponding author upon reasonable request.
